# AFLP Genome Scanning Reveals Divergent Selection in Natural Populations of *Liriodendron chinense* (Magnoliaceae) along a Latitudinal Transect

**DOI:** 10.3389/fpls.2016.00698

**Published:** 2016-05-26

**Authors:** Ai-Hong Yang, Na Wei, Peter W. Fritsch, Xiao-Hong Yao

**Affiliations:** ^1^Key Laboratory of Plant Germplasm Enhancement and Specialty Agriculture, Wuhan Botanical Garden, Chinese Academy of SciencesWuhan, China; ^2^Department of Ecology and Evolutionary Biology, University of MichiganAnn Arbor, MI, USA; ^3^Botanical Research Institute of TexasFort Worth, TX, USA

**Keywords:** outlier loci, environmental gradient, genome scan, local adaptation, Chinese Tulip Tree

## Abstract

Understanding adaptive genetic variation and its relation to environmental factors are important for understanding how plants adapt to climate change and for managing genetic resources. Genome scans for the loci exhibiting either notably high or low levels of population differentiation (outlier loci) provide one means of identifying genomic regions possibly associated with convergent or divergent selection. In this study, we combined Amplified Fragment Length Polymorphism (AFLP) genome scan and environmental association analysis to test for signals of natural selection in natural populations of *Liriodendron chinense* (Chinese Tulip Tree; Magnoliaceae) along a latitudinal transect. We genotyped 276 individuals from 11 populations of *L. chinense* using 987 AFLP markers. Both frequency-based (Dfdist and BayeScan) and correlation-based (MLM) methods were applied to detect outlier loci. Our analyses recovered both neutral and potentially adaptive genetic differentiation among populations of *L. chinense*. We found moderate genetic diversity within populations and high genetic differentiation among populations with reduced genetic diversity toward the periphery of the species ranges. Nine AFLP marker loci showed evidence of being outliers for population differentiation for both detection methods. Of these, six were strongly associated with at least one climate factor. Temperature, precipitation, and radiation were found to be three important factors influencing local adaptation of *L. chinense*. The outlier AFLP loci are likely not the target of natural selection, but the neighboring genes of these loci might be involved in local adaptation. Hence, these candidates should be validated by further studies.

## Introduction

Climate change has become a major threat to global biodiversity (Davis and Shaw, 2001; Parmesan, 2006). There is a growing evidence for shifts in species distributions and abundance in response to climate change (Parmesan, 2006; Matías and Jump, 2015). Species may be able to locally adapt to the new climatic conditions in current locations through genetic changes (Gienapp et al., 2008). Local adaptation of populations to climate has been revealed in a variety of plant species (González-Martínez et al., 2006; Savolainen et al., 2007; Coop et al., 2010; Hancock et al., 2011). Documenting the genetic basis of local adaptation governed by natural selection is important for understanding how plants adapt to their environment and respond to climatic changes.

Ecologists have studied local adaptation using reciprocal transplant experiments (e.g., Chartier et al., 2013). Population geneticists utilize genetic tools such as quantitative trait locus (QTL) mapping (Tanksley, 1993) and multiple-marker-based ‘neutrality’ tests (Storz, 2005) to study the genetic basis of local adaptation. For forest species, however, reciprocal transplant experiments and QTL mapping are not suitable for analysis of the adaptive genetic responses to climatic change due to their longer juvenile phase (Savolainen et al., 2007). Genome scans are an approach for identifying marker loci that are linked to selectively relevant target loci through ‘genetic hitchhiking’ (Luikart et al., 2003). Genome scans are widely used to detect signatures of local adaptation to environmental conditions (Bonin et al., 2007; Wood et al., 2008; Fischer et al., 2011; Buckley et al., 2012). In this method, large numbers of loci sampled throughout the genome are genotyped for many individuals sampled from two or more populations. Estimating population differentiation for all loci allows identification of ‘outlier loci’ whose level of differentiation among populations is either much greater or much less than that expected under neutral expectations (Lewontin and Krakauer, 1973; Storz, 2005; Foll and Gaggiotti, 2008; Nosil et al., 2009; Fischer et al., 2011). These outlier loci are assumed to be in linkage disequilibrium with genes involved in adaptive evolution due to genetic hitchhiking (Luikart et al., 2003; Schlötterer, 2003). Amplified Fragment Length Polymorphism (AFLP) markers (Vos et al., 1995) are popular for performing whole-genome scans for species whose genomes have not been sequenced due to their high polymorphism, ease of genotyping and analysis, and low cost (Mattersdorfer et al., 2012; Westberg et al., 2013). AFLP genome scans have been extensively used in studies of plant populations, e.g., *Howea* (Palmae; Savolainen et al., 2006), *Silene* (Caryophyllaceae; Minder and Widmer, 2008), *Mikania* (Asteraceae; Wang et al., 2012), and *Themeda* (Poaceae; Dell’Acqua et al., 2014).

A major limitation of genome scans is that they often detect false positives due to a departure from Hardy–Weinberg equilibrium and the assumption of the population structure model (Excoffier et al., 2009). Natural selection generates gradual changes in allele frequencies at outlier loci along environmental gradients (Manel et al., 2010). Thus, outlier loci can potentially be detected by a strong correlation between allele frequencies and environmental parameters (Coop et al., 2010). The correlative approach need not take population structure into account and can be used to seek confirmation of outlier loci following the identification of candidate loci with genome scan methods (Joost et al., 2007; Nunes et al., 2011; Bothwell et al., 2012; Henry and Russello, 2013).

*Liriodendron chinense* (Hemsl.) Sarg. (Magnoliaceae), commonly known as the Chinese Tulip Tree, is a self-incompatible, highly outcrossing and tall deciduous species (Hao et al., 1995). The species ranges widely in subtropical China and northern Vietnam, usually in mountains at elevations from ca. 450 to 1800 m (Hao et al., 1995), but is found only in scattered populations throughout its distribution. Its North American sister species, *L. tulipifera*, is similarly widespread in eastern Northern American broadleaf forests (Parks et al., 1994) but is much more common. Previous studies with microsatellite markers recovered a moderate level of within-population genetic diversity and strong genetic differentiation in *L. chinense* (Yang et al., 2016). The distribution pattern of *L. chinense* provides a suitable study system for examining divergent selection in natural populations along a latitudinal transect.

Outlier analysis has the potential to detect loci that have experienced both convergent and divergent selection. Strong selection for fitness conditions uniform among populations will prevent divergence by genetic drift, whereas strong selection pressures that are heterogeneous among populations will produce loci more diverged than expected by genetic drift. Divergent selection in natural populations has been well studied in a few model organisms in which candidate genes for traits of interest are known (e.g., Meyer et al., 2009). However, less is known about how changing climatic conditions will affect most species, including the tulip tree. This study combines an extensive sampling design with an AFLP genome scan and correlation analyses to test for signals of natural selection in natural populations of *L. chinense* along a latitudinal transect. Such information is essential for devising optimum management strategies for an *in situ* conservation program and the long-term survival of this species.

## Materials and Methods

### Plant Materials

**FIGURE 1:**
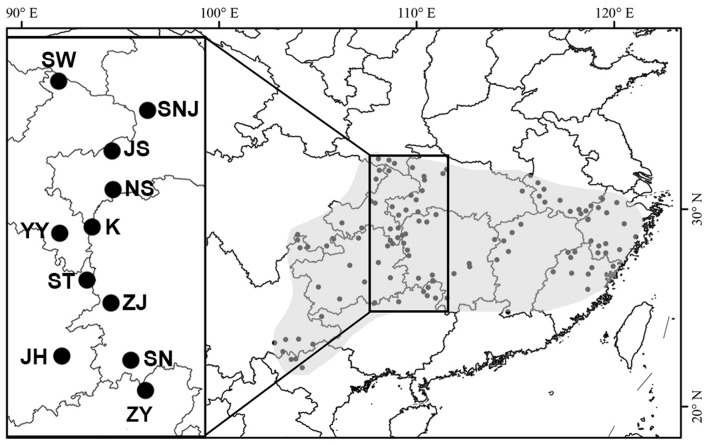
**Geographic location of 11 populations of *Liriodendron chinense* sampled along a latitudinal transect**.

In 2008, 1-year-old twigs with mature buds were collected from 276 individuals representing 11 populations of *L. chinense* along a rough latitudinal transect (**Table 1**; **Figure 1**). All twigs were wrapped with a damp paper towel and stored at 4°C until DNA extraction was carried out.

**Table 1:** Characteristics of 11 investigated natural populations of *Liriodendron chinense*.

Population	Location	Sample size	Latitude (N)	Longitude (E)
SNJ (C)	Shennongjia, Hubei province	28	31.401	110.405
JS (C)	Jianshi, Hubei province	20	30.713	109.680
NS (C)	Xuanen Hubei province	30	29.682	109.716
K (C)	Longshan, Hunan province	24	29.067	109.067
ZJ (C)	Zhijiang, Hunan province	28	27.597	109.638
SN (C)	Suining, Hunan province	29	26.448	110.108
YY (C)	Youyang, Chongqing	24	28.968	108.656
SW (M)	Chengkou, Chongqing	30	32.030	108.628
JH (C)	Jianghe, Guizhou province	30	26.497	108.690
ST (C)	Songtao, Guizhou province	20	28.157	109.319
ZY (M)	Ziyuan, Guangxi province	27	25.850	110.363

*C, central population; M, marginal population.*

### AFLP Genotyping

Genomic DNA was extracted from buds with the cetyltrimethylammonium bromide (CTAB) method (Doyle and Doyle, 1987). The quality and concentration of the DNA were determined by electrophoresis on 1% agarose gels with kDNA markers.

Amplified fragment length polymorphism analysis was carried out by following the method of Vos et al. (1995). Amplification was performed with 13 primer combinations, with each primer having three selective nucleotides (Supplementary Table S1). The *Eco*RI selective primers were labeled with fluorescent dye (6-FAM). Selective PCR products were sized against an internal standard (GeneScan-500 ROX, Applied Biosystems) on an ABI Genetic Analyser 3730 (Applied Biosystems) and analyzed by GeneMarker v2.2.0 (Applied Biosystems). Amplification products were scored for the presence or absence of bands, and non-discernible fragments (clearly identifiable bands with high peaks) were excluded from the analysis. The bands between 50 and 400 bp were scored as present (1) or absent (0). To test the repeatability of AFLP procedure, AFLP analysis was replicated twice for 12 randomly selected individuals, starting from DAN extraction down to capillary electrophoresis of the selective PCR products for each primer combination. AFLP loci with more than one score discrepancy for any primer combination were excluded from subsequent statistical analysis.

### Data Analyses

#### Genetic Diversity and Genetic Structure

The percentage of polymorphic loci (*PPL*) at 5% level corrected for the sample bias, expected heterozygosity within populations [*H*_S_, analogous to Nei’s (1973) unbiased expected gene diversity (*H*_eN_) assuming Hardy–Weinberg equilibrium], and population genetic differentiation (*F*_ST_) were computed with AFLP-SURV version 1.0 (Vekemans et al., 2002). To conduct genetic diversity comparison between central and marginal population, we defined the central and marginal populations of *L. chinense* by their geographic locations. In this study, the furthest north (SW) and south (ZM) population were referred as marginal population.

To quantify the degree of genetic differentiation among populations and infer the most appropriate number of subpopulations (*K*) for interpreting the data without prior information about the number of populations sampled and to which population each individual belonged, we used the individual-based population assignment test implemented in the program STRUCTURE (Pritchard et al., 2000). STRUCTURE analysis was conducted with only those markers that showed no indication of outlier behavior in BayeScan analysis. Ten replicates of each simulation from *K* = 1 to 20 were performed at 100,000 Markov’s chain Monte Carlo (MCMC) simulation by sampling after a burn-in period of 50,000 iterations. The admixture model and uncorrelated allele frequencies were chosen for the analysis. The most likely estimate of *K* was predicted from plots of *ad hoc* posterior probability models of *ΔK*. *ΔK* statistics are more appropriate than the highest Ln*Pr* (*X*|*K*) method to infer population number (Evanno et al., 2005). Population genetic differentiation was also estimated from the neutral data set (loci that showed no indication of outlier behavior in both methods) and the outlier data set (loci that showed an indication of outlier behavior in both methods).

#### Outlier Detection

Two complementary methods were applied to detect outlier loci of all populations in *L. chinense*. To reveal the impact of the variation in *N*_e_ among populations on the outlier detection, outlier tests were performed on the two marginal populations (SW, ZY) and two central populations (ZJ, NS) with similar population size. Firstly, Dfdist (Beaumont and Nichols, 1996) was used, which implements a hierarchical Bayesian approach based on summary statistics under Wright’s (1943) infinite island model at migration–drift equilibrium (Beaumont and Balding, 2004). Most common alleles (allele frequency > 99%) were discarded both for the estimation of the empirical multilocus *F*_ST_, and for simulations. The null distribution of *F*_ST_ (a ‘trimmed’ mean *F*_ST_) was obtained by removing 30% of the highest and lowest single-locus *F*_ST_ estimates according to the recommendation by Beaumont and Balding (2004). This ‘trimmed’ mean represents the ‘neutral’ *F*_ST_ values, supposedly uninfluenced by outlier loci. Coalescent simulations were performed to generate data sets with a null distribution based on 50,000 simulations and infinite island model. Outlier loci were detected by comparing empirical *F*_ST_ values for each locus (empirical distribution) against a null distribution of *F*_ST_ values expected from a neutral drift model (simulation distribution). The 0.995 or 0.005 quantiles were chosen to define an envelope within which 99% of the data points are expected to lie (Wang et al., 2012). Any loci occurring outside the expected range were considered as potential outliers.

Dfdist assumes that populations are at migration–drift equilibrium, which does not often occur in natural populations (Manel et al., 2009). The outlier loci identified by the Dfdist approach could be false positives (Herrera and Bazaga, 2008). To minimize the detection of false positives, we used BayeScan^1^, which is suitable for dominant markers such as those in AFLP and allows the estimation of the posterior probability of a given locus under selection (Foll and Gaggiotti, 2008). The Bayesian method assumes that allele frequencies within a population follow a Dirichlet distribution under Wright’s (1931) island model. The Bayesian method estimates population specific *F*_ST_ coefficients under a wide range of demographic scenarios and considers different amounts of genetic drift between populations (Foll and Gaggiotti, 2008). In addition, small numbers of samples can be analyzed by BayeScan with the risk of a low power, but with no particular risk of bias (Foll and Gaggiotti, 2008). A threshold value for determining loci under selection was evaluated in accordance with Jeffreys’ (1961) interpretation, i.e., log_10_ PO > 2.0 was considered as decisive evidence for selection. We employed a threshold of log_10_ PO > 2.0 for the rejection of the null hypothesis in each of the conducted tests. BayeScan analysis was conducted with a burn-in of 50,000 iterations, a thinning interval of 50, and a sample size of 10,000. The number of pilot runs was kept at 20 with length of 5,000 each.

#### Association with Climatic Parameters

Climate data, which included thermal, precipitation and total radiation records over the period 1971–2000 from more than 100 weather stations across the geographic range of *L. chinense*, were gathered from the China Ecosystem Research Network^2^ (in Chinese). Sixty-three climatic parameters, including mean temperature per year, monthly minimum and maximum temperatures, monthly average temperatures, monthly average precipitation, mean precipitation per year, total radiation each month, and mean radiation per year, were obtained from this database.

TASSEL (Bradbury et al., 2007) was used to identify significant associations between the population-level allele frequencies and climate factors. The basic assumption of this association analysis is that natural selection along an environmental gradient generates changes in allele frequencies at loci linked to selected genes (Schoville et al., 2012). Based on a *Q* model and the most stringent model of *Q* + *K*, *Q*-values (the membership coefficients for each individual) estimated from STRUCTURE for the neutral data set and/or kinship values (genetic covariance between pairs of individuals, *K*) calculated with SPAGeDi (Hardy and Vekemans, 2002) were used as covariates in mixed linear regression (MLM) analysis.

## Results

### Genetic Diversity and Genetic Structure

**FIGURE 2:**
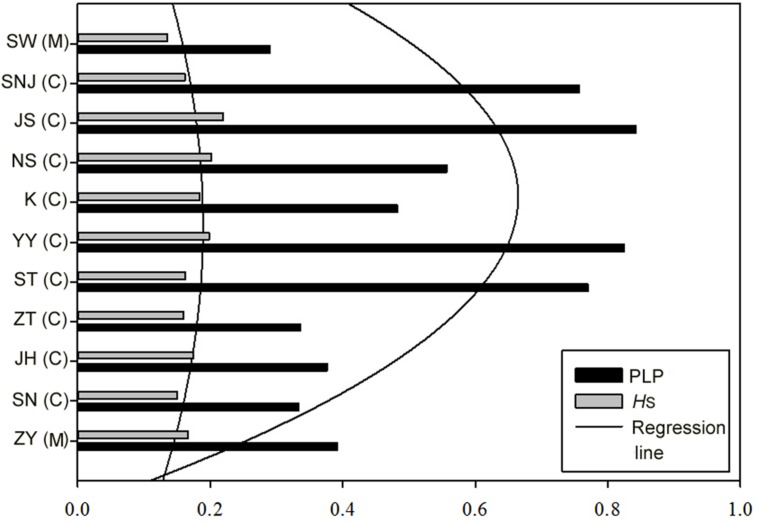
**Genetic diversity and trend line of 11 populations of *L. chinense* in the western region.** Populations are arranged by descending latitude. C in the bracket represents central population and M indicates marginal population. Fitted curves are polynomial quadratic. *H*: expected heterozygosity; *PLP*: proportion of polymorphic loci at the 5% level.

Thirteen primer pairs resolved a total of 987 unambiguous bands. The *PPL* per population ranged from 29.0 to 84.2%, with a mean value of 54.2%. The values of genetic diversity as estimated by expected heterozygosity (*H*) varied from 0.136 to 0.220, with a mean value of 0.174. The central populations had a higher *H* (0.200) than the average (0.174) across the 11 populations and was higher than that of marginal populations (**Figure 2**).

The overall value of *F*_ST_ based on all loci was 0.198. It was 0.171 and 0.571 for neutral and outlier loci, respectively (**Table 2**). Pairwise *F*_ST_ values at outlier loci between pairs of populations ranged from 0.169 to 0.899, whereas those at neutral loci ranged from 0.066 to 0.309 (Supplementary Table S2).

**Table 2:** Genetic diversity and genetic differentiation of 11 populations of *L. chinense* based on overall loci, natural loci, and outlier loci.

Loci	*N*	*H*_T_	*H*_S_	*F*_ST_
Overall loci	987	0.217	0.174	0.198
Neutral loci	932	0.206	0.171	0.171
Outlier loci	9	0.456	0.198	0.571

*N, number of loci scored; H, total gene diversity; H_S_, mean within-population expected heterozygosity; F_ST_, genetic differentiation.*

**FIGURE 3:**
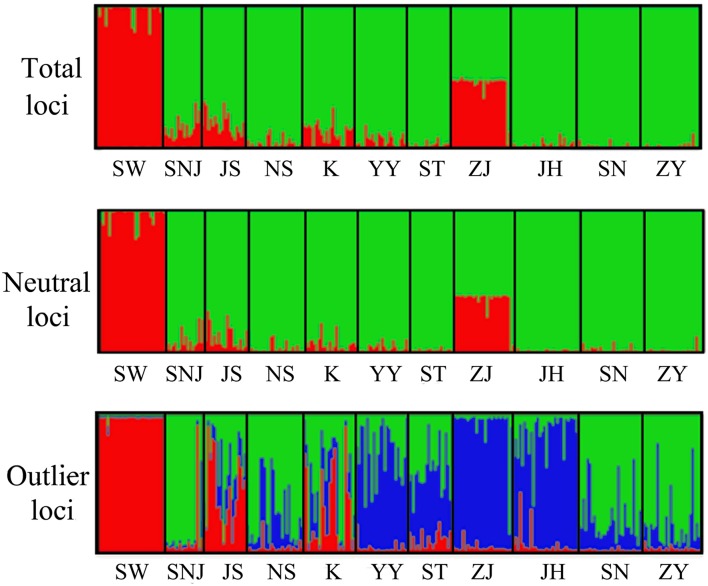
**Genetic relationships among the 11 populations studied with STRUCTURE based on total loci, neutral loci, and outlier loci.** Each individual is represented by one vertical column and populations are separated by vertical bars. Populations are arranged by descending latitude.

The plot of *ΔK* against a range of *K*-values showed the highest peak at *K* = 2. The *ΔK* statistics were found to be more appropriate than those from the highest Ln*Pr* (*X*|*K*) method to infer population number (Evanno et al., 2005). STRUCTURE analysis provided strong evidence for the presence of two independent populations (Clusters I and II). Cluster I only comprised population SW, and cluster II was mainly distributed among other populations. Individuals in ZJ populations were admixed and inherited from two different ancestors; this was also the case for the neutral data set (**Figure 3**).

### Outlier Analyses

**FIGURE 4:**
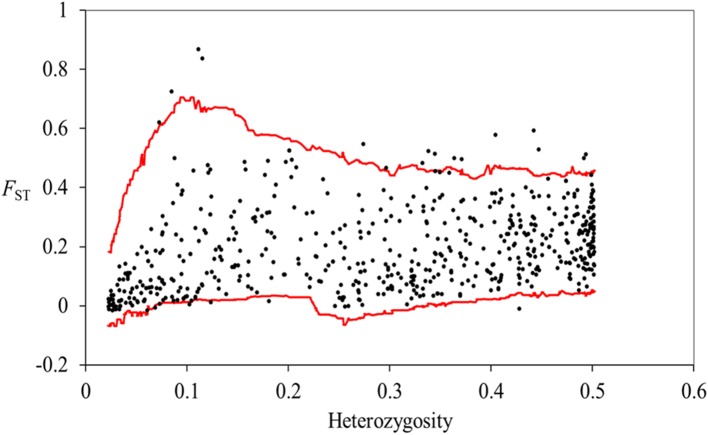
**Results of FDIST analysis.** The red line indicates the 99% upper and lower confidence levels; loci beyond these levels are identified as outlier loci.

**FIGURE 5:**
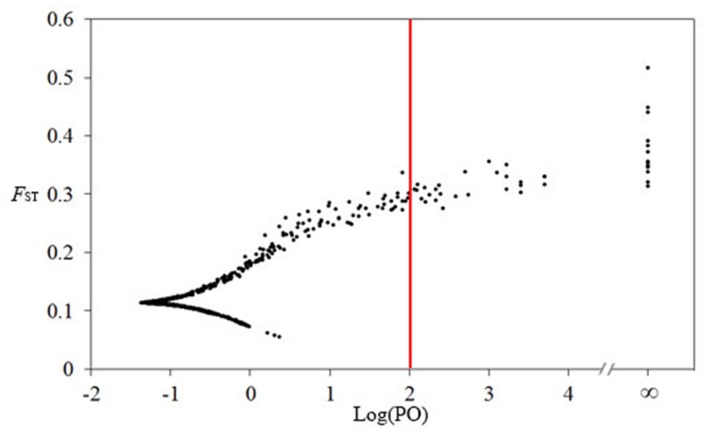
**BayeScan plots of 987 amplified fragment length polymorphism (AFLP) loci in 11 populations of *L. chinense.*** The vertical red line is the threshold [Log(PO) = 2] used for identifying outlier loci. Dots that fall to the right of the threshold line are identified as outlier loci.

In the Dfdist analysis, 21 outlier loci (2.13% of AFLPs) were identified as being outside of the 99% null distribution. Among these, 16 exhibited more divergence and five exhibited less divergence than the majority of loci, consistent with divergent and convergent selection, respectively (**Figure 4**). BayeScan analysis identified 43 high-differentiation loci at a threshold of log_10_ PO > 2.0 (posterior probabilities higher than 0.99), corresponding to 4.36% of all loci (**Figure 5**), all of which exhibited increased differentiation. A total of 55 loci were identified by either Dfdist or BayeScan analyses, with nine loci were detected by both methods. For two marginal populations and two central populations, Dfdist analysis identified 9 and 10 outlier loci, and BayeScan analysis detected four and nine outlier loci, respectively. One and two outlier loci were detected by both methods for two marginal populations and two central populations, respectively.

### Genetic Variation Associated with Climate Parameters

The GLM test based on a *Q* model yielded 114 loci (11.6%) that exhibited a significant association with one or more climatic factors at the significance threshold set to 1.0E-10. Eleven of 21 loci (52.4%) detected with Dfdist were significantly associated with at least one climate factor. Twenty-eight of 43 loci (65.1%) detected by BayeScan were significantly associated with at least one climate factor (**Supplementary Figure S1**). Six of nine loci (66.7%) detected by both Dfdist and BayeScan were significantly associated with at least one climatic factor (**Supplementary Figure S2** and **Table 3**). Based on the most stringent model of *Q* + *K*, only one locus (896) was found to be associated with the climate factors. This locus was also detected by Dfdist, BayeScan, and association analysis with the *Q* model.

**Table 3:** Six loci related to local adaptation identified by all the three methods.

Locus name	Primer pairs	Fragment length
442	F: E-AGG/M-CAA	142
492	F: E-AGG/M-CAA	227
493	F: E-AGG/M-CAA	228
530	G: E-AGC/M-CGA	103
570	G: E-AGC/M-CGA	188
896	L: E-ACA/M-CAA	140

## Discussion

### Genetic Diversity and Genetic Structure

*Liriodendron chinense* showed an intermediate level of intra-population genetic diversity (*H*s = 0.174) as compared with AFLP genetic diversity observed in other endangered tree species (e.g., *Malus sylvestris*: *H*_eN_ = 0.225, Coart et al., 2003; *Berchemiella wilsonii* var. *pubipetiolata*: *H*_eN_ = 0.163, Kang et al., 2007; *Eucommia ulmoides*: *H*_eN_ = 0.174, Yao et al., 2012). A similarly moderate level of gene diversity was also found in 29 range-wide populations of *L. chinense* with microsatellite markers (*H*_E_ = 0.570). The genetic structure revealed by AFLPs (*F*_ST_ = 0.198) is also largely concordant with the pattern revealed by microsatellites in *L. chinense* (Yang et al., 2016). Stronger genetic structure based on non-neutral markers was also revealed in other studies and explained through the absence of gene flow facilitating the establishment of local adaptations (e.g., Midamegbe et al., 2011). The pattern of genetic diversity found in *L. chinense* most likely reflects historical demography and biological traits of this species. Despite historical fragmentation due to montane glaciation and subsequent climatic oscillation, the high longevity of the species may buffer the species against the loss of genetic diversity and allow the long-term maintenance of genetic variation in such an ancient species. In addition, limited pollen and seed dispersal among relict populations might contribute to the extraordinarily high diversity among populations found in this species.

The central–marginal hypothesis predicts lower genetic diversity and higher genetic differentiation in marginal populations of a species’ range as compared with those in the central regions (Sagarin et al., 2006; Eckert et al., 2008). In our study, populations of *L. chinense* in the middle-latitude regions tend to have higher genetic diversity than those of low latitude and high latitude regions, which supports the central–marginal hypothesis. This is consistent with evidence from microsatellite markers, in which higher genetic variation was observed in the core populations as compared with southern marginal populations at large geographical scales (Yang et al., 2016). From field observations, we found that the peripheral populations are smaller and more fragmented. Peripheral populations with small population size may suffer from reduced gene flow and strong genetic drift, leading to lower genetic diversity and higher genetic differentiation than those in central populations (Eckert et al., 2008).

### Adaptation along a Latitudinal Transect

In order to identify genes and genomic regions potentially related to local adaptation, the 987 AFLP markers were screened for the footprints of divergent selection among 11 populations along a latitudinal transect. Simulation studies show that the BayeScan test is more efficient than the Dfdist test in the identification of outlier loci with dominant markers (Pérez-Figueroa et al., 2010). In our study, 2.1 and 4.4% of the total number of AFLP fragments assayed were identified as putatively positive outlier adaptive loci with Dfdist and BayeScan, respectively. As found in other studies (e.g., Moore et al., 2014), the BayeScan test revealed a much higher number of outlier loci than did the Dfdist test. The overall outlier detection rates are similar to those reported in other genomic scans based on AFLP markers. For instance, 3% in lake whitefish (Campbell and Bernatchez, 2004) and 3–4% in lizards (Nunes et al., 2011) are typically reported as departing from the neutral expectation.

The recovery of false positives can occur in population genome scans. The statistical power of genome scan studies may be affected by genotyping errors, poor genome coverage of AFLP markers, statistical departures from the model assumptions, complex population structure and demographic history (Nei and Maruyama, 1975; Bonin et al., 2006; Excoffier et al., 2009; Meyer et al., 2009), all of which have been thoroughly considered in the previous reviews (Luikart et al., 2003; Beaumont and Balding, 2004; Storz, 2005; Bonin et al., 2006, 2007; Caballero et al., 2008; Excoffier et al., 2009). Excoffier et al. (2009) showed outlier analysis scan was sensitive to population model assumptions by comparing island models and hierarchical models. The perception of outliers can change drastically depending on the assumptions used to model population structure (Nei and Maruyama, 1975; Excoffier et al., 2009). Both Dfdist and BayeScan used island model as the null hypothesis, however, island model was an unrealistic population structure model as most populations might violate the assumption of this model. In the present study, variation in effective population sizes (*N*_e_) between the central and marginal populations suggests *L. chinense* possibly departs from island model assumption and lead to many false positives (Nei and Maruyama, 1975). Lower *N*_e_ observed in the marginal populations leads to a wider distribution of *F*_ST_ values among loci since stronger genetic drift may leave genomic signatures that mimic selection. Thus, genetic drift rather than natural selection may produce the ‘outlier loci’ (false positives), which was supported by the fact that fewer outlier loci identified in two marginal populations compared to two central populations when pairwise comparisons were made. Hidden population structure causing correlated allele frequencies can also lead to a high false-positive rate in the detection of selection (Excoffier et al., 2009; Narum and Hess, 2011). The SW population is divergent from other populations as shown in **Figure 3**, which suggests the existing population structure may have a major effect on outlier detection. However, nine loci identified by both methods were also detected even when the SW population was excluded in the analysis (data not shown). In addition, demographic history can produce patterns similar to positive selection, as in cases of severe bottlenecks, allele surfing during population expansion, secondary contact, and isolation by distance (Schoville et al., 2012; Lotterhos and Whitlock, 2014).

Reducing the false positives as much as possible remains a critical element in genome scanning (Thornton and Jensen, 2007). To reduce the number of false positives, we used both Dfdist and BayeScan, two approaches that differ in algorithms and assumptions (Paris et al., 2010; Wang et al., 2012), and we used stringent significance thresholds in both analyses. We used a conservative approach in estimating the number of loci involved in local adaptation by excluding outliers specific to one or the other analysis. This approach recovered nine loci that diverge from neutrality and are thus likely involved in local adaptation. Nevertheless, it is inherently difficult to identify which outlier loci are false positives (Narum and Hess, 2011). The outlier loci must be confirmed through further analysis, including sequencing and molecular functional analysis of neighboring genes.

Because the outlier-detection methods used here (Dfdist and BayeScan) do not directly integrate tests for specific selection pressures (e.g., environmental factors) that cause selection (Schoville et al., 2012), we also used an environmental correlation strategy to identify loci underlying local adaptation. Environmental association analysis is commonly used to detect loci that have been subject to natural selection (Coop et al., 2010; Eckert et al., 2010). In our study, 65.1% outlier loci identified by BayeScan are strongly associated with climatic factors, and 52.4% outlier loci identified by Dfdist possess a significant association with at least one climatic factor. When applying GLM analysis to the AFLP data set and taking population structure into account, we found that six out of the nine loci identified by both Dfdist and BayeScan were also found to be correlated with the climatic factors. Although strong gene flow, distance effects, and historical demography can create allele frequencies that are correlated with environment parameters solely through neutral processes (Manel et al., 2003; Eckert et al., 2010), suggesting that the results of genome scans should be interpreted with caution, when considered as a whole, our analyses suggest that at least six loci are locally adapted and thus reveal evidence of divergent selection among populations.

In the present study, temperature, precipitation, and radiation were identified as the three major drivers of allele distributions along the latitudinal transect (Supplementary Table S3). There is increasing evidence that populations distributed over altitudinal and latitudinal gradients are differentially adapted to spatially variable environmental conditions (Saxe et al., 2001; Manel et al., 2010), such as temperature (Jump et al., 2006; Kooyers and Olsen, 2012), precipitation (Manel et al., 2010), and radiation (Parisod and Pascal-Antoine, 2008). The prominent role of temperature, precipitation, and radiation in driving plant adaptation was also revealed in previous studies (St. Clair et al., 2005; Richardson et al., 2009; Manel et al., 2010). For instance, using population genome scan methods, Richardson et al. (2009) found that several AFLP outlier loci were strongly associated with temperature and precipitation in *Pinus monticola*. Kooyers and Olsen (2012) found that allele frequencies at cyanogenesis genes were associated with minimum winter temperature in *Trifolium repens*. Through ecological niche modeling, precipitation and temperature of the coldest quarter were found to be the main factors contributing to geographic distribution of *L. chinense* (Yang et al., 2016). In addition, He and Hao (1998) emphasized the role of relative humidity of February to April and average temperature of January to April in determining seed set in *L. chinense.* Among the six outlier AFLPs, three were common to the temperature, precipitation, and radiation partition. The documented overlap of physiological mechanisms involved in adaptation to different climatic factors was also inferred in other studies (e.g., Prunier et al., 2011).

## Conclusion

The results of this study revealed high levels of genetic differentiation among populations and moderate levels of genetic diversity within populations of *L. chinense*. Such information can be useful in the protection of this endangered species. A set of nine outlier loci that departed from neutral expectations were revealed in 11 populations of *L. chinense* along a latitudinal transect. Six of these were also found to be strongly associated with at least one of the three climatic factors studied. Precipitation from winter to spring, temperature, and radiation in early spring were identified as key environmental factors that contribute to the adaptive differentiation of this species. This study paves the way for identifying the molecular basis of local adaptation in *L. chinense*. Further studies are needed to characterize the outlier AFLP bands, identify their genomic locations and neighboring genes, and validate the underlying genes involved in local adaptation in *L. chinense*. Pinpointing these genes is made possible by the availability of the EST (expressed sequence tags) database of *L. tulipifera*, a sister species of *L. chinense* (Liang et al., 2008).

## Author Contributions

X-HY and NW conceived the ideas; A-HY collected the data; A-HY analyzed the data. The manuscript was written by A-HY, PF, and X-HY.

## Conflict of Interest Statement

The authors declare that the research was conducted in the absence of any commercial or financial relationships that could be construed as a potential conflict of interest.

## Footnotes

1 ibe.ch/software/BayeScan/index.html

2 http://www.cern.ac.cn/0index

## Supplementary material

FIGURE S1 **Venn diagrams summarizing the number of candidate loci detected by BayeScan, DFDIST, and TASSEL**.

FIGURE S2 **Polyacrylamide gel images of PCR products of AFLP markers for the possible candidate loci**.
