# Supplementary figures and images for: AFLP Genome Scanning Reveals Divergent Selection in Natural Populations of Liriodendron chinense (Magnoliaceae) along a Latitudinal Transect
Source: Front Plant Sci. 2016 May 26;7:698. doi: 10.3389/fpls.2016.00698 (PMC4880593; doi:10.3389/fpls.2016.00698)

Supporting materials

Figure S1.

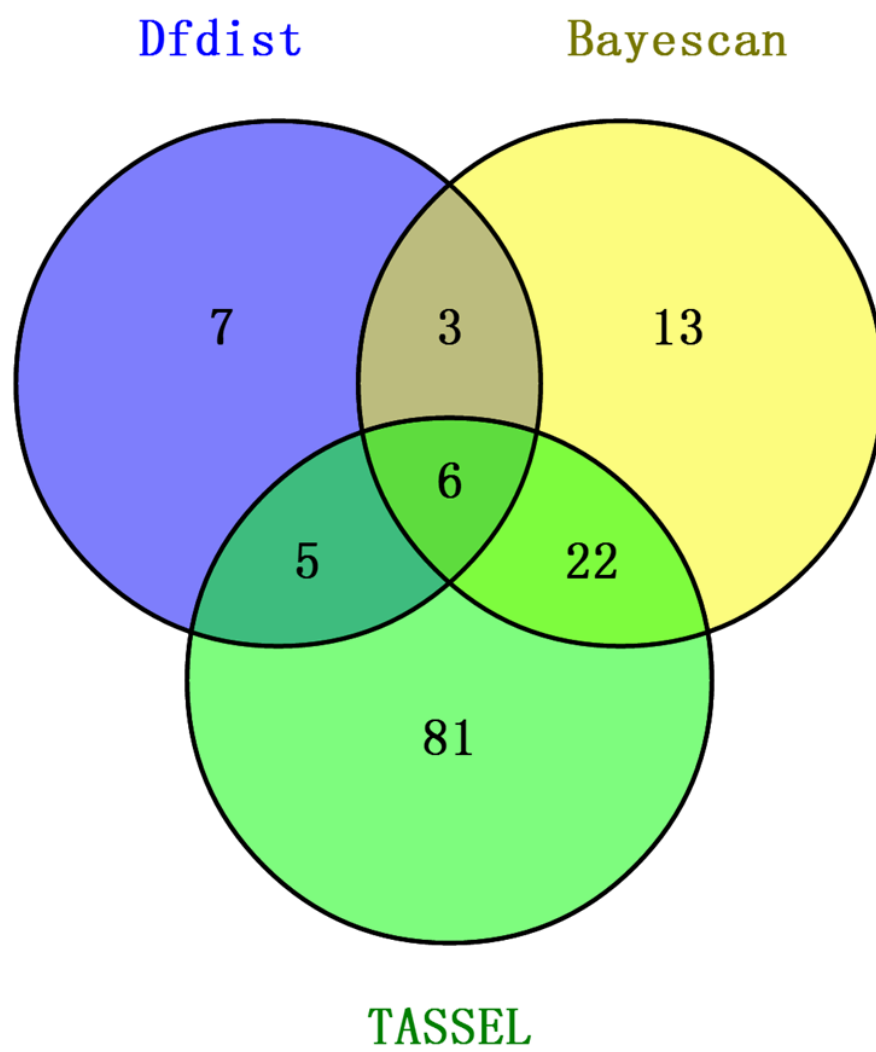

**Fig S2**

**a**

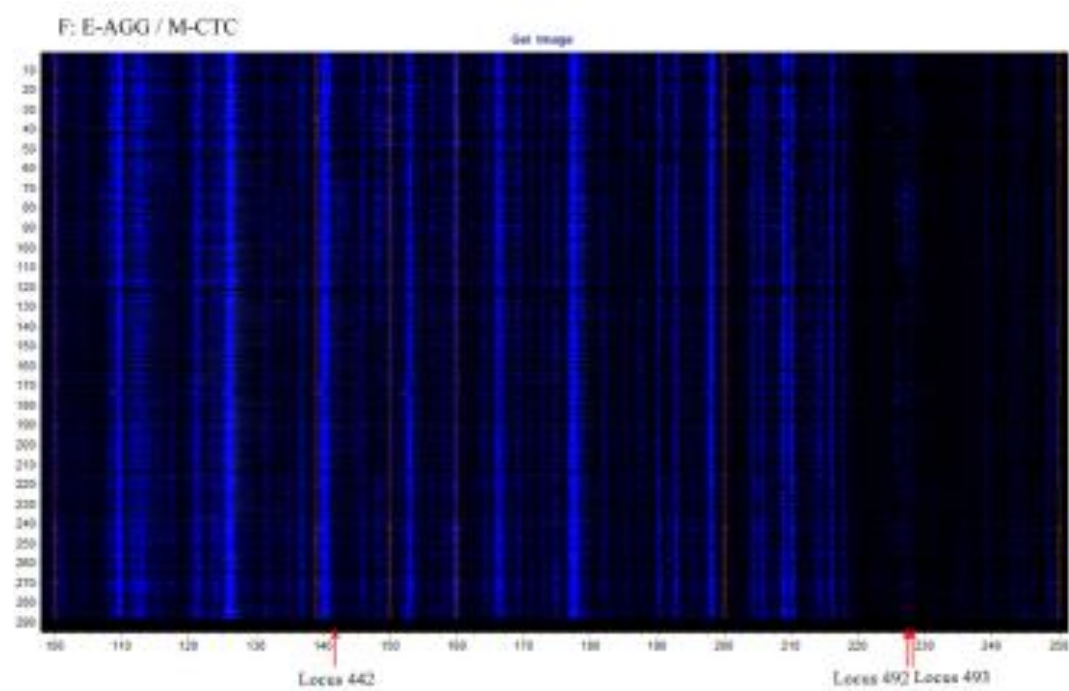

**b**

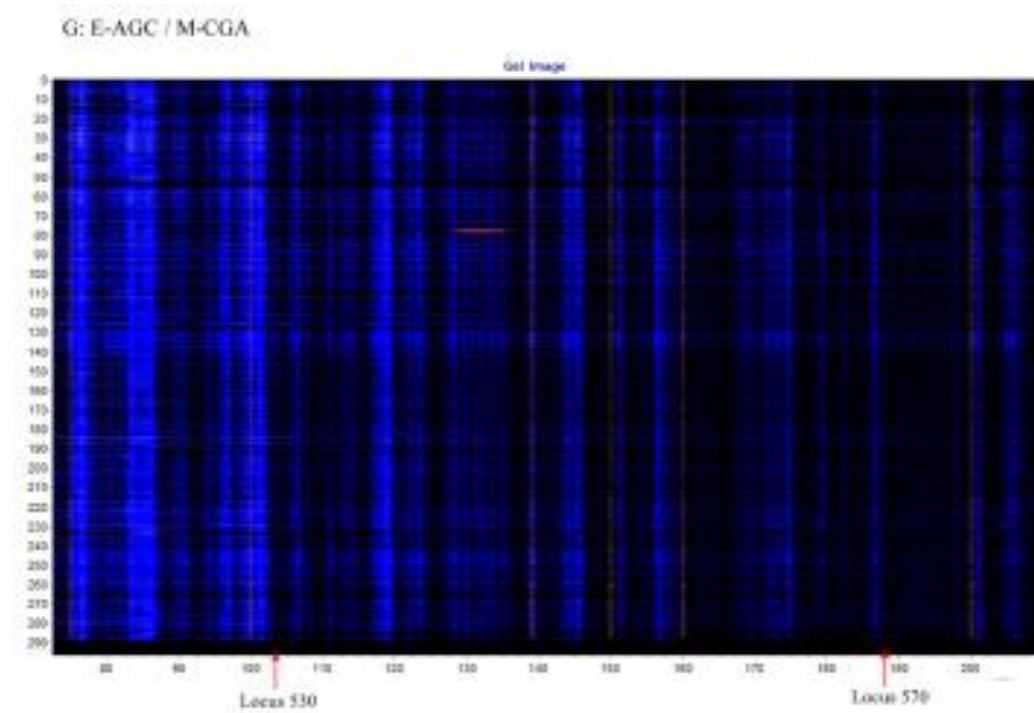

c

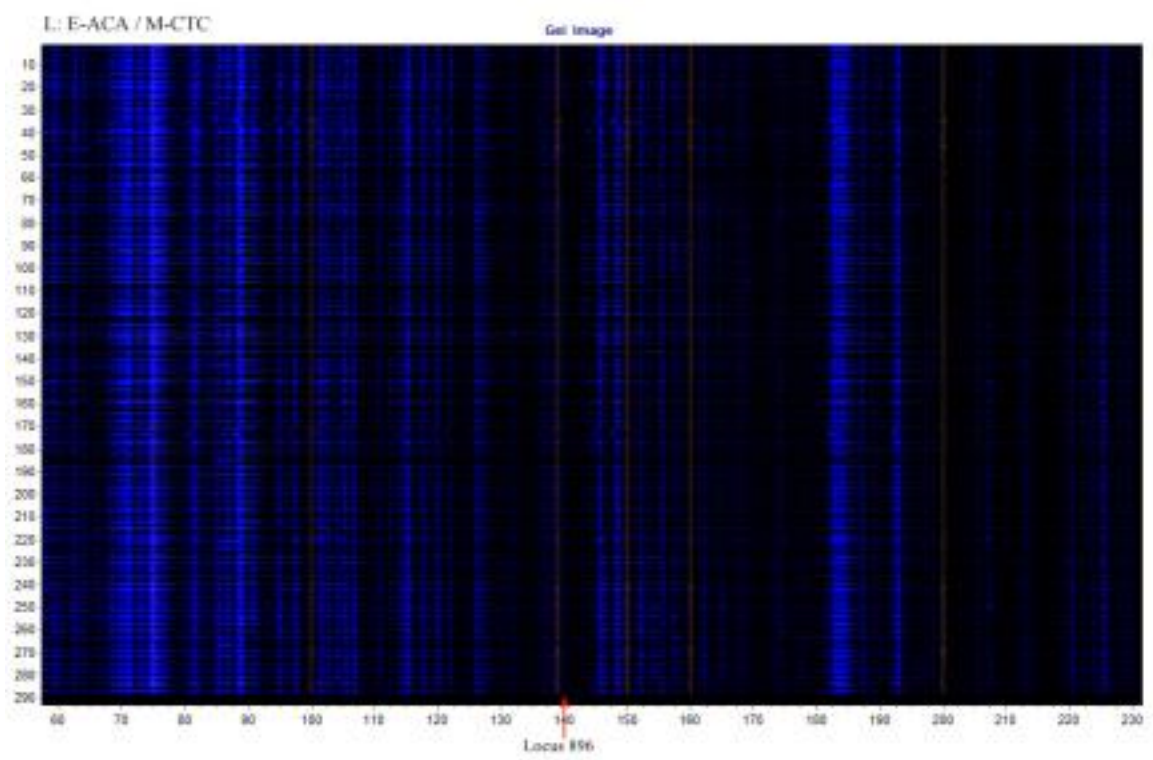

Supplement: FIGURE S1 — Venn diagrams summarizing the number of candidate loci detected by BayeScan, DFDIST, and TASSEL. [file Image_1.PDF]
