# Supplementary material for: AFLP Genome Scanning Reveals Divergent Selection in Natural Populations of Liriodendron chinense (Magnoliaceae) along a Latitudinal Transect
Source: Front Plant Sci. 2016 May 26;7:698. doi: 10.3389/fpls.2016.00698 (PMC4880593; doi:10.3389/fpls.2016.00698)
Supplement: Supplementary file 2 [file Table_1.DOCX]

**Supporting materials**

**Table S1.** Sequences of adapters and primers for AFLP analysis.

| Sequence properties | Sequence code | Components of sequence or primer pairs |
| --- | --- | --- |
| Adaptor | *Eco*RI adaptor | 5′-CTCGTAGACTGCGTACC-3′ |
|  |  | 5′-AATTGGTACGCAGTCTAC-3′ |
|  | *Mse*I adaptor | 5′-GACGATGAGTCCTGAG-3′ |
|  |  | 5′-TACTCAGGACTCAT-3′ |
| Pre-amplification primers | EA00 | 5′-GTAGACTGCGTACCAATTCA-3′ |
|  | MC00 | 5′-GACGATGAGTCCTGAGTAAC-3′ |
| Selective amplification primer-pairs | A | E-ACT / M-CCA (HEX) |
|  | B | E-ACA / M-CAC (FAM) |
|  | C | E-AAC/ M-CAG (HEX) |
|  | D | E-ACA / M-CTT (FAM) |
|  | E | E-ACT / M-CTC (HEX) |
|  | F | E-AGG / M-CTC (FAM) |
|  | G | E-AGC / M-CGA (FAM) |
|  | H | E-AGG / C-CAT (FAM) |
|  | I | E-AGG / M-CAG (FAM) |
|  | J | E-ACT / M-CAG (HEX) |
|  | K | E-ACC / C-CAT (FAM) |
|  | L | E-ACA / M-CTC (FAM) |
|  | M | E-AAC / C-CAT (HEX) |

The fluorescent dye for each primer pair are in brackets.
