# Supplementary material for: AFLP Genome Scanning Reveals Divergent Selection in Natural Populations of Liriodendron chinense (Magnoliaceae) along a Latitudinal Transect
Source: Front Plant Sci. 2016 May 26;7:698. doi: 10.3389/fpls.2016.00698 (PMC4880593; doi:10.3389/fpls.2016.00698)
Supplement: Supplementary file 3 [file Table_2.DOCX]

**Supporting materials**

**Table S2.** Pairwise population genetic differentiation (*F*_ST_) based on neutral (below diagonal) and outlier (above diagonal) loci.

|  | JS | SNJ | SW | K | NS | ZY | SN | JH | YY | ST | ZJ |
| --- | --- | --- | --- | --- | --- | --- | --- | --- | --- | --- | --- |
| JS | - | 0.314 | 0.614 | 0.169 | 0.391 | 0.424 | 0.569 | 0.445 | 0.345 | 0.375 | 0.534 |
| SNJ | 0.135 | - | 0.728 | 0.368 | 0.563 | 0.373 | 0.611 | 0.626 | 0.575 | 0.558 | 0.632 |
| SW | 0.179 | 0.279 | - | 0.670 | 0.826 | 0.796 | 0.899 | 0.762 | 0.775 | 0.754 | 0.762 |
| K | 0.069 | 0.181 | 0.225 | - | 0.562 | 0.470 | 0.543 | 0.487 | 0.410 | 0.544 | 0.388 |
| NS | 0.073 | 0.181 | 0.244 | 0.128 | - | 0.459 | 0.626 | 0.594 | 0.434 | 0.442 | 0.734 |
| ZY | 0.091 | 0.206 | 0.257 | 0.129 | 0.123 | - | 0.521 | 0.463 | 0.319 | 0.329 | 0.553 |
| SN | 0.169 | 0.266 | 0.309 | 0.201 | 0.176 | 0.183 | - | 0.687 | 0.492 | 0.725 | 0.722 |
| JH | 0.103 | 0.227 | 0.278 | 0.133 | 0.133 | 0.133 | 0.195 | - | 0.216 | 0.354 | 0.355 |
| YY | 0.066 | 0.175 | 0.227 | 0.097 | 0.078 | 0.092 | 0.155 | 0.089 | - | 0.326 | 0.464 |
| ST | 0.091 | 0.213 | 0.265 | 0.111 | 0.120 | 0.114 | 0.195 | 0.130 | 0.071 | - | 0.596 |
| ZJ | 0.163 | 0.246 | 0.304 | 0.174 | 0.195 | 0.199 | 0.256 | 0.181 | 0.159 | 0.182 | - |
