# Supplementary material for: AFLP Genome Scanning Reveals Divergent Selection in Natural Populations of Liriodendron chinense (Magnoliaceae) along a Latitudinal Transect
Source: Front Plant Sci. 2016 May 26;7:698. doi: 10.3389/fpls.2016.00698 (PMC4880593; doi:10.3389/fpls.2016.00698)
Supplement: Supplementary file 4 [file Table_3.DOCX]

**Supporting materials**

**Table S3.** Association between AFLP loci frequency and climatic variables as detected by mixed linear regression (MLM) analysis.

| Locus name | Climate factor | *p*-value |
| --- | --- | --- |
| 442 | Prec_7 | 4.51E-12 |
| 442 | Prec_9 | 4.69E-13 |
| 442 | Prec_10 | 4.18E-13 |
| 442 | Radia_2 | 1.77E-11 |
| 442 | Radia_3 | 1.82E-16 |
| 442 | Radia_4 | 7.16E-22 |
| 442 | Radia_5 | 5.73E-18 |
| 442 | Radia_6 | 1.96E-13 |
| 442 | Tmax_1 | 2.28E-13 |
| 442 | Tmax_2 | 1.19E-10 |
| 442 | Tmax_3 | 4.37E-12 |
| 442 | Tmax_4 | 4.35E-11 |
| 442 | Tmax_5 | 9.00E-11 |
| 442 | Tmax_9 | 4.05E-13 |
| 442 | Tmax_10 | 2.53E-13 |
| 442 | Tmax_11 | 1.84E-14 |
| 442 | Tmax_12 | 7.59E-15 |
| 442 | Tmean_1 | 6.85E-14 |
| 442 | Tmean_2 | 2.50E-11 |
| 442 | Tmean_3 | 1.23E-13 |
| 442 | Tmean_4 | 8.08E-13 |
| 442 | Tmean_5 | 6.27E-12 |
| 442 | Tmean_6 | 1.21E-10 |
| 442 | Tmean_10 | 7.15E-12 |
| 442 | Tmean_11 | 3.07E-13 |
| 442 | Tmean_12 | 5.10E-14 |
| 442 | Tmean_average | 9.77E-12 |
| 442 | Tmin_1 | 3.29E-14 |
| 442 | Tmin_2 | 1.26E-12 |
| 442 | Tmin_3 | 1.68E-14 |
| 442 | Tmin_4 | 9.26E-14 |
| 442 | Tmin_5 | 1.69E-12 |
| 442 | Tmin_6 | 1.94E-11 |
| 442 | Tmin_9 | 1.18E-10 |
| 442 | Tmin_10 | 7.48E-11 |
| 442 | Tmin_11 | 1.94E-12 |
| 442 | Tmin_12 | 2.55E-13 |
| 492 | Prec_1 | 3.54E-10 |
| 492 | Prec_2 | 2.57E-11 |
| 492 | Prec_3 | 2.75E-14 |
| 492 | Prec_8 | 4.74E-14 |
| 492 | Prec_12 | 1.33E-14 |
| 492 | Prec_average | 1.19E-14 |
| 492 | Radia_1 | 1.76E-17 |
| 492 | Radia_2 | 2.65E-12 |
| 492 | Radia_5 | 1.35E-11 |
| 492 | Radia_6 | 4.76E-15 |
| 492 | Radia_10 | 1.68E-12 |
| 492 | Radia_11 | 1.10E-13 |
| 492 | Radia_12 | 5.71E-16 |
| 492 | Radia_average | 4.48E-17 |
| 493 | Prec_1 | 3.90E-27 |
| 493 | Prec_2 | 2.43E-27 |
| 493 | Prec_3 | 1.70E-27 |
| 493 | Prec_4 | 9.63E-26 |
| 493 | Prec_5 | 1.25E-20 |
| 493 | Prec_12 | 3.60E-28 |
| 493 | Prec_average | 1.33E-11 |
| 493 | Radia_7 | 9.68E-21 |
| 493 | Radia_8 | 4.67E-11 |
| 493 | Radia_9 | 1.24E-16 |
| 493 | Radia_10 | 2.04E-20 |
| 493 | Radia_11 | 1.31E-17 |
| 493 | Radia_12 | 4.49E-16 |
| 493 | Radia_average | 2.11E-13 |
| 530 | Prec_7 | 1.38E-26 |
| 530 | Prec_8 | 4.07E-17 |
| 530 | Prec_9 | 5.13E-18 |
| 530 | Prec_average | 3.79E-15 |
| 530 | Radia_1 | 6.73E-13 |
| 530 | Radia_2 | 7.60E-16 |
| 530 | Radia_3 | 5.38E-15 |
| 530 | Radia_4 | 2.13E-13 |
| 530 | Radia_5 | 2.45E-13 |
| 530 | Radia_6 | 4.61E-11 |
| 530 | Tmax_1 | 1.66E-10 |
| 530 | Tmax_10 | 2.87E-12 |
| 530 | Tmax_11 | 2.02E-12 |
| 530 | Tmax_12 | 1.78E-11 |
| 530 | Tmax_3 | 9.50E-11 |
| 530 | Tmax_4 | 1.10E-11 |
| 530 | Tmax_5 | 3.51E-12 |
| 530 | Tmax_6 | 4.83E-12 |
| 530 | Tmax_7 | 3.92E-13 |
| 530 | Tmax_8 | 1.38E-13 |
| 530 | Tmax_9 | 2.23E-13 |
| 530 | Tmean_1 | 1.93E-12 |
| 530 | Tmean_10 | 1.10E-13 |
| 530 | Tmean_11 | 1.03E-13 |
| 530 | Tmean_12 | 2.84E-13 |
| 530 | Tmean_2 | 5.86E-11 |
| 530 | Tmean_3 | 2.01E-12 |
| 530 | Tmean_4 | 3.40E-13 |
| 530 | Tmean_5 | 8.05E-14 |
| 530 | Tmean_6 | 1.36E-13 |
| 530 | Tmean_7 | 5.09E-14 |
| 530 | Tmean_8 | 5.77E-14 |
| 530 | Tmean_9 | 1.57E-11 |
| 530 | Tmean_average | 3.12E-13 |
| 530 | Tmin_1 | 1.41E-13 |
| 530 | Tmin_2 | 1.19E-11 |
| 530 | Tmin_3 | 1.50E-13 |
| 530 | Tmin_4 | 3.09E-14 |
| 530 | Tmin_5 | 8.04E-15 |
| 530 | Tmin_6 | 7.95E-15 |
| 530 | Tmin_7 | 1.25E-14 |
| 530 | Tmin_8 | 2.89E-15 |
| 530 | Tmin_9 | 9.46E-15 |
| 530 | Tmin_10 | 1.51E-14 |
| 530 | Tmin_11 | 8.09E-15 |
| 530 | Tmin_12 | 3.31E-14 |
| 570 | Prec_7 | 1.91E-12 |
| 570 | Prec_9 | 4.17E-12 |
| 896 | Prec_1 | 2.21E-13 |
| 896 | Prec_2 | 6.74E-13 |
| 896 | Prec_3 | 3.41E-14 |
| 896 | Prec_4 | 6.06E-11 |
| 896 | Prec_5 | 2.02E-14 |
| 896 | Prec_6 | 8.27E-14 |
| 896 | Prec_7 | 1.73E-15 |
| 896 | Prec_8 | 4.60E-19 |
| 896 | Prec_9 | 3.75E-22 |
| 896 | Prec_12 | 4.86E-10 |
| 896 | Prec_average | 1.60E-17 |
| 896 | Radia_1 | 3.47E-11 |
| 896 | Radia_4 | 1.21E-10 |
| 896 | Radia_5 | 1.36E-10 |
| 896 | Radia_6 | 2.14E-14 |
| 896 | Radia_9 | 2.80E-11 |
| 896 | Radia_10 | 4.55E-12 |
| 896 | Radia_11 | 3.28E-12 |
| 896 | Radia_12 | 1.90E-12 |

The number 1 to 12 indicates January to December.
